# Association Between Monocyte‐to‐High‐Density Lipoprotein Ratio and Diabetic Nephropathy: A Systematic Review and Meta‐Analysis

**DOI:** 10.1155/bmri/1097476

**Published:** 2026-03-28

**Authors:** Ibrahim Anyass Goumboundi, Justice Afrifa, Richard K. D. Ephraim

**Affiliations:** ^1^ Department of Medical Laboratory Science, University of Cape Coast, Cape Coast, Ghana, ucc.edu.gh; ^2^ Kidney Research Initiative Ghana, Cape Coast, Ghana

**Keywords:** diabetic nephropathy, inflammation, monocyte-to-HDL ratio

## Abstract

**Background:**

Diabetic nephropathy (DN) is a major microvascular complication of diabetes mellitus and a leading cause of chronic kidney disease worldwide. Inflammation and dyslipidemia play central roles in its pathogenesis. The monocyte‐to‐high‐density lipoprotein cholesterol ratio (MHR) has emerged as a composite biomarker integrating proinflammatory and anti‐inflammatory pathways, but its association with DN has not been systematically synthesized.

**Objective:**

This systematic review and meta‐analysis evaluated the association between MHR and DN and quantified the differences in MHR between diabetic patients with and without nephropathy.

**Methods:**

A comprehensive literature search of PubMed, the Cochrane Library, and Google Scholar was conducted from inception to 2025. Observational studies reporting MHR in adult diabetes mellitus patients with and without DN were included. Standardized mean differences (SMDs) with 95% confidence intervals (CIs) were pooled using random effects models. Heterogeneity was assessed using the *I* ^2^ statistic. Risk of bias was evaluated using the Newcastle–Ottawa Scale, and publication bias was examined using funnel plots and Egger′s test.

**Results:**

(9) observational studies comprising 1797 participants were included. Across most studies, MHR was significantly higher in patients with DN compared with those without DN. The pooled analysis demonstrated a significantly elevated MHR in the DN group, with a positive SMD favoring DN. Substantial between‐study heterogeneity was observed. Risk of bias was low to moderate in most studies. Funnel plot inspection and Egger′s regression test did not indicate significant publication bias (*p* = 0.486).

**Conclusions:**

This meta‐analysis demonstrates that MHR is significantly elevated in patients with DN, supporting its role as a marker of inflammation–lipid imbalance in diabetic kidney disease. Given its availability from routine laboratory parameters, MHR may serve as a practical adjunct biomarker for DN risk stratification.

## 1. Introduction

Diabetic nephropathy (DN), a chronic microvascular complication of diabetes mellitus, is a leading cause of chronic kidney disease and end‐stage renal failure worldwide. It is clinically characterized by increasing albuminuria and declining renal function, often progressing silently until advanced stages [1]. Traditional diagnostic markers such as urinary albumin‐to‐creatinine ratio (UACR) and estimated glomerular filtration rate (eGFR) are useful for disease monitoring but may not fully capture early inflammatory and metabolic disturbances that contribute to DN pathogenesis [2]. This limitation emphasizes the need for accessible biomarkers that reflect underlying pathophysiology and improve risk stratification in diabetic populations.

Inflammation and oxidative stress are central to the development and progression of DN [3], contributing to endothelial dysfunction, glomerular damage, and renal fibrosis. Monocytes, as circulating proinflammatory cells, represent opposing forces within systemic inflammatory pathways [4, 5]. The monocyte‐to‐high‐density lipoprotein cholesterol ratio (MHR) integrates these two components into a single metric that may better reflect the balance between pro‐ and anti‐inflammatory activity compared with monocyte or HDL levels alone. Recent regional evidence from Türkey also shows that specific inflammatory mediators such as Maresin‐1 and CHI3L1 exhibit differential expression and correlate with renal dysfunction in DN, underscoring the complexity of inflammatory pathways in DN [6].

Clinical studies indicate that MHR is elevated in patients with DN compared to diabetes mellitus patients without nephropathy and healthy controls, and it may correlate with markers of renal impairment. Higher MHR values have been observed in DN groups compared with non‐DN groups, suggesting that increased systemic inflammation and altered lipid metabolism are associated with kidney disease in diabetes mellitus [7]. Some research also reports that MHR correlates with albuminuria and renal function indicators like eGFR, indicating potential utility as a marker of disease severity and progression [8].

Although individual observational studies report associations between MHR and DN, results vary in magnitude and in the strength of correlation with renal outcomes. There has been no comprehensive synthesis of these findings to date, which limits understanding of how consistently MHR relates to DN across different populations and study designs. This systematic review and meta‐analysis is aimed at quantitatively summarizing available evidence on the association between MHR and DN and assessing the robustness of this association.

## 2. Method

### 2.1. Protocol Registration

This systematic review and meta‐analysis was conducted in accordance with the Preferred Reporting Items for Systematic Reviews and Meta‐Analyses (PRISMA) guidelines [9]. The protocol has been registered in the International Prospective Register of Systematic Reviews (PROSPERO), with the Registration Number PROSPERO 2026 CRD420261289087.

### 2.2. Eligibility Criteria

#### 2.2.1. Population

Studies involving adult patients with diabetes mellitus were eligible. The study population included individuals with DN and without DN (non‐DN).

#### 2.2.2. Exposure

The exposure of interest was the MHR, reported as a continuous variable.

#### 2.2.3. Comparator

Comparisons were made between diabetes mellitus patients with nephropathy and those without nephropathy.

#### 2.2.4. Outcomes

The primary outcome was the mean MHR, compared between diabetes mellitus patients with and without nephropathy. Minor variations in diagnostic criteria were observed; most studies defined DN using comparable albuminuria‐based thresholds (e.g., UACR ≥ 30 mg/g or equivalent), often combined with eGFR or proteinuria, in line with established clinical guidelines.

#### 2.2.5. Study Designs

Observational studies, including cross‐sectional, retrospective, and prospective cohort studies, were included. Case reports, reviews, editorials, and animal studies were excluded.

### 2.3. Information Sources and Search Strategy

A comprehensive literature search was conducted in PubMed, the Cochrane Library, and Google Scholar from database inception to the most recent update. Search strategies combined Medical Subject Headings and free‐text terms related to the MHR, DN, and diabetes mellitus. In addition, the reference lists of all included studies were manually screened to identify potentially relevant articles not captured by the database searches: for PubMed ((“neutrophil to HDL ratio” OR “NHR” OR “lymphocyte to HDL ratio” OR “LHR” OR “monocyte to HDL ratio” OR “MHR” OR “white cell to HDL ratio”) AND (“diabetic nephropathy” OR “diabetic kidney disease” OR “DKD” OR “microalbuminuria” OR “macroalbuminuria” OR “albuminuria” OR “Diabetic Nephropathies”[MeSH]) AND (“diabetes” OR “type 1 diabetes” OR “type 2 diabetes” OR “T1DM” OR “T2DM” OR “Diabetes Mellitus”[MeSH])).

### 2.4. Study Selection

All retrieved records were independently screened by two reviewers (I.A.G. and J.A.) at the title and abstract level to assess eligibility. Full‐text articles of potentially relevant studies were subsequently reviewed independently by the same reviewers using the predefined inclusion and exclusion criteria. Disagreements during the screening and selection process were resolved through discussion and consensus. When consensus could not be reached, a third reviewer (R.K.D.E.) was consulted to make the final decision.

### 2.5. Data Extraction

Data were independently extracted using a standardized data collection form. Extracted information included the first author and year of publication, study design and country, sample size, mean and standard deviation of MHR in DN and nonnephropathy groups, diagnostic criteria for DN, and relevant statistical measures reported by the original studies. When MHR data were reported as medians with interquartile ranges, established statistical methods were applied to estimate corresponding mean values and standard deviations to enable inclusion in pooled analyses.

### 2.6. Risk of Bias Assessment

Risk of bias was assessed using the Newcastle–Ottawa Scale (NOS) for observational studies. Each study was evaluated across the domains of selection (maximum four stars), comparability (maximum two stars), and outcome or exposure assessment (maximum three stars), with total scores ranging from 0 to 9. Studies scoring 7–9 points were considered low risk of bias, 5–6 points moderate risk, and fewer than 5 points high risk.

### 2.7. Statistical Analysis

The primary effect measure was the standardized mean difference with 95% confidence intervals, calculated from the obtained mean MHR values and standard deviations in DN and non‐DN groups. Random effects models were applied to account for between‐study heterogeneity, and pooled estimates were presented using forest plots. Heterogeneity was assessed using Cochran′s *Q* test and quantified with the *I* ^2^ statistic. In addition to standardized effects, pooled mean MHR values were estimated separately for DN and non‐DN groups using random effects models to enhance clinical interpretability. Publication bias was evaluated using funnel plots and Egger′s regression test, with *p* < 0.05 indicating potential small‐study effects. All analyses were performed using R software (Version 4.3.1). Meta‐analyses were conducted using the meta and metafor packages. Forest plots and heterogeneity statistics were generated using the meta package, while publication bias analyses were performed using the metafor package.

## 3. Results

### 3.1. Study Selection

**Figure 1:**
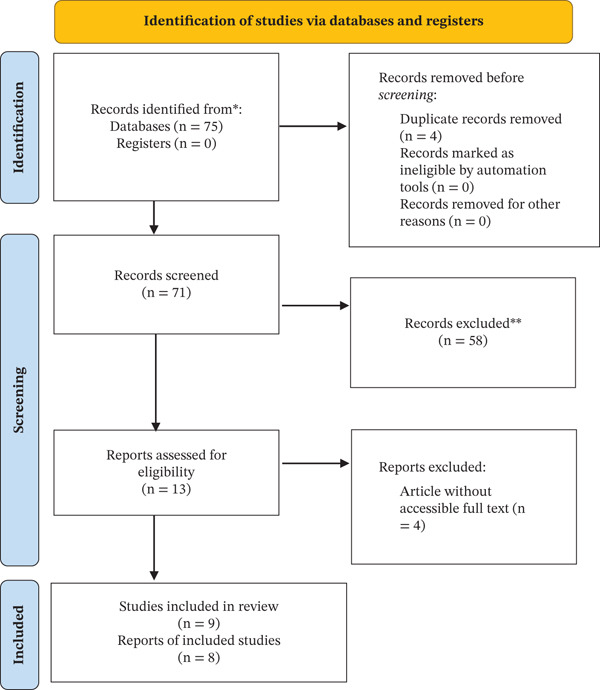
Flowchart of study selection.

The systematic search identified eligible studies assessing the association between the MHR and DN. After screening and full‐text assessment, nine observational studies met the inclusion criteria and were included in the quantitative synthesis. The study selection process is summarized in Figure 1.

### 3.2. Study Characteristics

The included studies, published between 2016 and 2025, comprised a total of 1797 adult participants with diabetes mellitus, predominantly Type 2 diabetes mellitus. Study designs included retrospective, cross‐sectional, and prospective observational studies. DN was defined using standard clinical criteria, most commonly based on albuminuria, proteinuria, and/or reduced eGFR. Study characteristics are summarized in Table 1.

**Table 1:** Characteristics of included studies.

Author	Country	Design	Sample size (*N*)	Diabetes type	Definition of DN
Sonmezoz and Yilmaz [10]	Türkey	Retrospective study	360	T2DM	UACR ≥ 30 mg/g
Karatas et al. [11]	Türkey	Cross‐sectional study	220	T2DM	UACR > 300 mg/g
Yang et al. [12]	China	Cross‐sectional retrospective study	159	T2DM	UACR > 300 mg/g
Yashilha et al. [13]	India	Prospective observational study	120	T2DM	UACR > 300 mg/g
Onalan [7]	Türkey	Retrospective study	262	T2DM	Proteinuria and/or creatinine levels above 1.2 mg/dL
Efe [8]	Türkey	Retrospective study	190	Patients living with diabetes mellitus	UACR ≥ 30 and/or eGFR < 60 mL/min
Ozer et al. [14]	Türkey	Retrospective study	152	Patients living with diabetes mellitus	24‐h urine albuminuria levels above 30 mg/day
Kahraman et al. [15]	Türkey	Retrospective study	134	T2DM	24‐h UAE > 300 mg/day
Veena and Rani Aravapalli [16]	India	Retrospective study	100	DN	NR

### 3.3. Study‐Level Comparison of MHR

Across most included studies, mean MHR values were higher in patients with DN compared with those without nephropathy (Table 2). While the magnitude of difference varied, the direction of association was generally consistent, with only one study reporting no statistically significant between‐group difference.

**Table 2:** MHR in diabetic nephropathy and non‐diabetic nephropathy groups.

Author	Group (*n*)	Mean±SD	p value
Sonmezoz and Yilmaz [10]	DN (55)	9.63 ± 3.51	0.687
	Non‐DN (206)	9.57 ± 4.30	
Karatas et al. [11]	DN (77)	17.6 ± 6.15	< 0.0001
	Non‐DN (72)	8.2 ± 3.04	
Yang et al. [12]	DN (43)	11.57 ± 7.82	0.029
	Non‐DN (43)	9.70 ± 4.24	
Yashilha et al. [13]	DN (30)	13.38 ± 4.83	< 0.0001
	Non‐DN (30)	9.08 ± 2.26	
Onalan [7]	DN (60)	17.1 ± 7.9	< 0.001
	Non‐DN (202)	10.3 ± 3.3	
Efe [8]	DN (85)	16.2 ± 5.5	0.037
	Non‐DN (105)	14.3 ± 4.5	
Ozer et al. [14]	DN (68)	15.25 ± 8.5	< 0.001
	Non‐DN (84)	12.12 ± 7.22	
Kahraman et al. [15]	DN (41)	14.6 ± 9.3	0.005
	Non‐DN (43)	11.2 ± 4.9	
Veena and Rani Aravapalli [16]	DN (100)	14.62 ± 7.4	0.0009

### 3.4. Pooled Mean MHR Estimates

**Figure 2:**
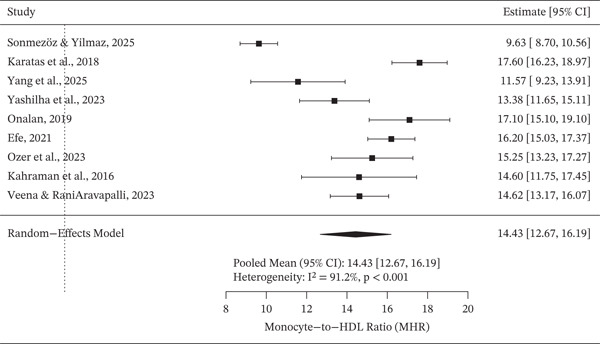
A forest plot displaying the pooled mean of MHR value among DN groups.

**Figure 3:**
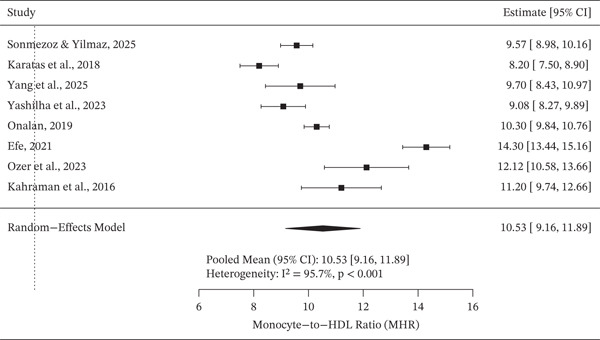
A forest plot displaying the pooled mean of MHR value among non‐DN groups.

Random effects meta‐analysis demonstrated elevated pooled mean MHR values among patients with DN (Figure 2). Substantial between‐study heterogeneity was observed, indicating variability in study populations and clinical characteristics. In contrast, pooled mean MHR values among diabetes mellitus patients without nephropathy were lower overall (Figure 3), although heterogeneity across studies remained evident.

### 3.5. Meta‐Analysis of Standardized Mean Differences

**Figure 4:**
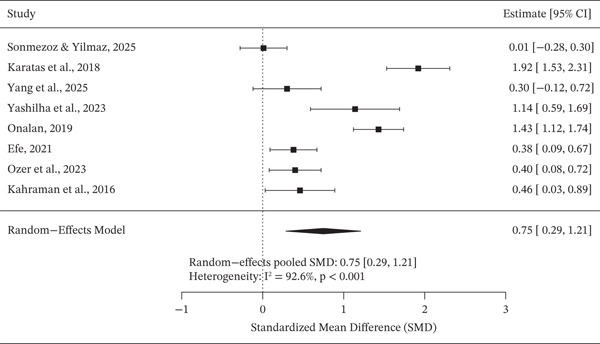
A forest plot showing the SMD of the MHR value between DN and non‐DN groups in DM patients.

The pooled standardized mean difference analysis showed that MHR was significantly higher in patients with DN compared with those without nephropathy (SMD = 0.75; 95% CI: 0.29–1.21; *I* ^2^ = 92.5*%*) (Figure 4).

### 3.6. Risk of Bias Assessment

NOS scores across the included studies ranged from 4 to 8 out of 9. Three studies were rated as low risk of bias, five as moderate risk, and one as high risk. Selection and outcome or exposure assessment was generally adequate, while limited adjustment for confounding factors accounted for lower comparability scores in several studies. Overall, the methodological quality of the included studies was acceptable for quantitative synthesis (Table 3).

**Table 3:** Risk of bias assessment.

Study	Selection	Comparability	Outcome/exposure	NOS score
Efe, 2021	★★★	★	★★	6/9
Onalan, 2019	★★★	★★	★	6/9
Karatas et al., 2018	★★★★	★★	★★	8/9
Kahraman et al., 2016	★★★	★	★	5/9
Ozer et al., 2023	★★	★	★★	5/9
Sonmezoz and Yilmaz, 2025	★★★★	★★	★★	8/9
Veena and Rani Aravapalli, 2023	★★	—	★★	4/9
Yang et al., 2025	★★★	★★	★★	7/9
Yashilha et al., 2023	★★★★	★★	★★	8/9

### 3.7. Publication Bias

**Figure 5:**
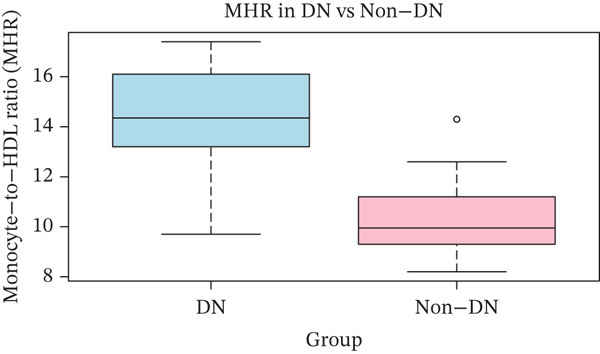
Box plot displaying the comparison of MHR value in the DN and non‐DN groups.

**Figure 6:**
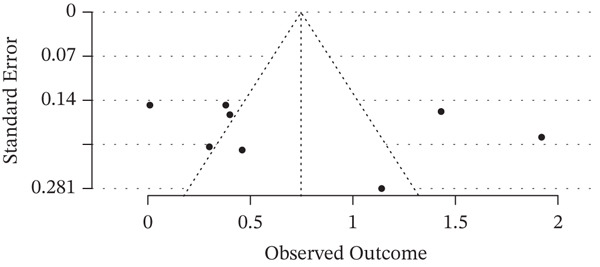
Funnel plot for publication bias of MHR in diabetic nephropathy.

Visual inspection of the funnel plot did not reveal marked asymmetry (Figure 5). Egger′s regression test did not suggest significant small‐study effects (*p* = 0.4861) (Figure 6).

## 4. Discussion

The present meta‐analysis found that the MHR is significantly elevated in patients with DN compared to those without. This result is biologically plausible given the roles of inflammation and dyslipidemia in DN. Chronic hyperglycemia promotes activation of monocytes/macrophages, which infiltrate the kidney and secrete proinflammatory cytokines such as TNF‐*α* and IL‐1*β* that directly damage glomerular and tubular structures [17]. In contrast, HDL normally has antioxidant and anti‐inflammatory effects that protect the vasculature and renal tissue [18]. Thus, a high MHR indicating elevated monocyte‐driven inflammation relative to HDL′s protective capacity reflects a shift toward a proinflammatory state. Our pooled estimate confirms that this imbalance is consistently observed in DN. In support of this, Arabi et al. showed that MHR “reflects monocyte‐driven inflammation and HDL′s anti‐inflammatory properties” and is strongly associated with diabetes and kidney disease risk [19]. In other words, MHR integrates the burden of inflammatory cells with lipid dysregulation. The higher MHR in DN patients suggests that the combined effect of increased monocyte count and reduced HDL magnifies oxidative stress and inflammatory signaling in the diabetic kidney, promoting glomerulosclerosis and renal dysfunction.

Our findings align with previous studies of MHR in diabetes‐related complications. Tang et al. observed that MHR was significantly higher in patients with diabetic retinopathy than in diabetes patients without retinopathy or healthy controls [20], implying that MHR tracks with microvascular injury beyond the kidney. Similarly, Karatas et al. reported higher MHR in diabetes mellitus patients with nephropathy versus normoalbuminuric diabetic patients, with MHR correlating positively with albuminuria [11]. In a recent Chinese cohort, Yang et al. found that MHR levels rose across albuminuria grades and each quartile increase in MHR was associated with higher odds of DN [12]. These studies support our pooled result that elevated MHR is a hallmark of DN. In contrast, other diabetes complications show mixed results: One investigation found no difference in MHR between diabetes with and without peripheral neuropathy [21]. Beyond microvascular disease, MHR has also been linked to macrovascular risk in diabetes: Higher MHR predicts subclinical atherosclerosis and cardiovascular events [22]. Thus, the literature consistently indicates that MHR is elevated in inflammatory diabetic complications such as DN and retinopathy, but its association may vary by complication type.

Several pathophysiological mechanisms could link a high MHR to DN progression. Diabetic milieu induces upregulation of chemokines and adhesion molecules in the kidney, which selectively recruit circulating monocytes into glomeruli and interstitium [17]. These monocytes differentiate into macrophages that release TNF‐*α*, IL‐6, and other mediators causing endothelial dysfunction, increased glomerular permeability, mesangial expansion, and fibrosis [17]. Thus, increased monocytes may directly drive renal inflammation. Concurrently, reduced HDL levels amplify damage by diminishing HDL′s protective roles. HDL normally limits monocyte recruitment and activation, scavenges oxidized lipids, and preserves endothelial and podocyte function [23]. In diabetes mellitus, HDL becomes glycated and functionally impaired, further compromising reverse cholesterol transport. A low HDL state permits lipid deposition in glomeruli and enhances oxidative stress, accelerating sclerosis [24]. In sum, a high MHR signifies both an abundance of proinflammatory monocytes and a lack of anti‐inflammatory HDL. This dual effect likely potentiates intrarenal inflammation, oxidative injury, and consequent DN progression.

### 4.1. Comparison of MHR With Other Relevant Inflammatory and Lipid‐Based Ratios

Several hematological and inflammatory indices have been studied as biomarkers in DN. The neutrophil‐to‐lymphocyte ratio (NLR) and platelet‐to‐lymphocyte ratio (PLR) have been frequently evaluated as markers of systemic inflammation in diabetes and its microvascular complications. A large meta‐analysis of inflammatory biomarkers in DN found that NLR was significantly higher in patients with microalbuminuria and macroalbuminuria compared with normoalbuminuric diabetics and that higher NLR was associated with increased odds of DN and higher inflammatory burden, underscoring NLR′s potential as an inflammatory indicator in DN pathology. PLR and the systemic immune‐inflammation index (SII) were observed to be elevated in some comparisons but with less consistent predictive value across studies [25].

NLR reflects the balance between innate neutrophil‐mediated immune activation and adaptive lymphocyte‐mediated regulatory control, and its elevation has been linked with increased inflammatory activity, endothelial dysfunction, and microvascular injury in chronic diabetes states. This has been evidenced by its association with DN progression and mortality in several observational cohorts and aggregated analyses [26].

PLR, which incorporates platelet activation along with lymphocyte suppression, has been reported to be higher in DN and other inflammatory states. However, in pooled models, the association of PLR with DN did not reach statistical significance in some meta‐analytic comparisons, suggesting its role may be less specific or influenced by cofactors such as platelet activation in comorbid conditions [25].

SII, constructed from neutrophils, lymphocytes, and platelets, attempts to comprehensively capture inflammatory and immune responses but has shown variable performance depending on population and DN stage in existing studies, with heterogeneity limiting definitive conclusions about its clinical utility [25].

Compared with these indices, MHR uniquely integrates an immune cell subset (monocytes) directly implicated in renal inflammatory processes with HDL cholesterol, which has established anti‐inflammatory and antioxidative effects. Monocyte infiltration into the kidney and subsequent macrophage differentiation play central roles in glomerulosclerosis and tubulointerstitial damage, while HDL exerts endothelial protection and limits monocyte recruitment and oxidative stress. By combining these opposing pathways, MHR may more specifically reflect the pathophysiology of DN than ratios based solely on leukocyte subsets or platelets.

From a clinical perspective, MHR has potential utility as a biomarker in DN but also limitations. It can be readily calculated from routine blood counts and lipid profiles, making it an inexpensive and widely available index of systemic inflammation and dyslipidemia. Arabi et al. noted that “MHR is a feasible and accessible biomarker of inflammation and lipid dysregulation” [19]. In practice, a high MHR might prompt closer monitoring of renal function or cardiovascular risk. Moreover, MHR may have some advantages over single parameters: As a composite index, it captures the balance between damaging monocytes and protective HDL [19]. However, MHR is nonspecific. Its elevation reflects general inflammatory states or dyslipidemia, which can be caused by infection, obesity, or other comorbidities. Thus, MHR alone cannot diagnose DN.

Future research should address these gaps. Ideally, longitudinal cohort studies would test whether baseline MHR predicts the onset or progression of DN independently of known risk factors. Interventional studies could explore whether targeting inflammation or raising HDL (e.g., with statins or lifestyle changes) reduces MHR and favorably impacts renal outcomes. Mechanistic studies are also needed; for instance, renal biopsy or imaging studies could examine monocyte/macrophage infiltration and HDL composition in patients stratified by MHR. Additionally, research in Type 1 diabetes and in underrepresented populations would clarify generalizability. Such work would determine whether MHR is merely a correlate of DN or a modifiable risk marker. In summary, our meta‐analysis supports the concept that an elevated MHR reflecting increased inflammation and altered lipid metabolism is associated with DN. MHR shows promise as a low‐cost biomarker, but its clinical application awaits further validation.

### 4.2. Strengths and Limitations

The current study has some strengths and limitations. Our systematic pooling of multiple studies strengthens the evidence for the MHR–DN association. However, the observational nature of the data, variable DN definitions, and residual confounding remain important limitations. We also noted high statistical heterogeneity across pooled analyses and variability in diagnostic criteria for DN among included studies, which may influence effect estimates and comparability. The predominance of retrospective observational designs and regional/geographic clustering of studies particularly those from Türkey may limit generalizability to broader populations. Despite these limitations, the use of pooled estimates and a comprehensive literature review are key strengths that advance understanding of MHR′s role in DN.

## 5. Conclusions

The consistent finding of higher MHR in DN patients across studies suggests that MHR captures important inflammatory and metabolic dimensions of DN. Given its accessibility, MHR could become part of a panel of DN risk indicators. Future prospective and mechanistic studies are warranted to establish its prognostic value and to elucidate the underlying biology.

## Funding

No funding was received for this manuscript.

## Ethics Statement

The authors have nothing to report.

## Consent

The authors have nothing to report.

## Conflicts of Interest

The authors declare no conflicts of interest.

## Data Availability

The datasets analyzed during the current study are derived from previously published articles that are publicly available and have been appropriately cited in this manuscript. The compiled dataset used for the meta‐analysis is available from the corresponding author on reasonable request.
